# An Efficient Strategy to Induce and Maintain *In Vitro* Human T Cells Specific for Autologous Non-Small Cell Lung Carcinoma

**DOI:** 10.1371/journal.pone.0012014

**Published:** 2010-08-09

**Authors:** Glenda Canderan, Paola Gruarin, Daniela Montagna, Raffaella Fontana, Giulio Melloni, Catia Traversari, Paolo Dellabona, Giulia Casorati

**Affiliations:** 1 Experimental Immunology Unit, Division of Immunology, Transplantation and Infectious Diseases, San Raffaele Scientific Institute, Milan, Italy; 2 Laboratory of Immunology, Department of Pediatrics, University of Pavia, Pavia, Italy; 3 Cancer Gene Therapy Unit, Division of Molecular Oncology, San Raffaele Scientific Institute, Milan, Italy; 4 Department of Thoracic Surgery, San Raffaele Scientific Institute, Milan, Italy; 5 MolMed S.P.A., Milan, Italy; Centre de Recherche Public de la Santé (CRP-Santé), Luxembourg

## Abstract

**Background:**

The efficient expansion *in vitro* of cytolytic CD8^+^ T cells (CTLs) specific for autologous tumors is crucial both for basic and translational aspects of tumor immunology. We investigated strategies to generate CTLs specific for autologous Non-Small Cell Lung Carcinoma (NSCLC), the most frequent tumor in mankind, using circulating lymphocytes.

**Principal Findings:**

Classic Mixed Lymphocyte Tumor Cultures with NSCLC cells consistently failed to induce tumor-specific CTLs. Cross-presentation *in vitro* of irradiated NSCLC cells by autologous dendritic cells, by contrast, induced specific CTL lines from which we obtained a high number of tumor-specific T cell clones (TCCs). The TCCs displayed a limited TCR diversity, suggesting an origin from few tumor-specific T cell precursors, while their TCR molecular fingerprints were detected in the patient's tumor infiltrating lymphocytes, implying a role in the spontaneous anti-tumor response. Grafting NSCLC-specific TCR into primary allogeneic T cells by lentiviral vectors expressing human V-mouse C chimeric TCRα/β chains overcame the growth limits of these TCCs. The resulting, rapidly expanding CD4^+^ and CD8^+^ T cell lines stably expressed the grafted chimeric TCR and specifically recognized the original NSCLC.

**Conclusions:**

This study defines a strategy to efficiently induce and propagate *in vitro* T cells specific for NSCLC starting from autologous peripheral blood lymphocytes.

## Introduction

The growth of transformed cells is controlled to some extent by the immune system, a phenomenon termed tumor immunesurveillance (reviewed in [1]).

Compelling evidence for the involvement of CD8^+^ cytotoxic T lymphocytes (CTLs) in tumor immunosurveillance has emerged in recent years with the cloning of genes encoding tumor associated antigens (TAAs), and the subsequent molecular identification of tumor-specific T cell epitopes [2], [3], [4]. The efficient induction *in vitro* of CTLs specific for autologous tumors is critical for relevant aspects of tumor immunology, including the generation of cellular probes to exploit in the discovery of new TAAs or the expansion of effector cells for adoptive immunotherapy approaches. Non-Small Cell Lung Carcinoma (NSCLC) is the leading cause of cancer death worldwide and responds poorly to current therapies (reviewed in [5]). Systemic immunotherapy can be envisaged as an attractive innovative therapeutic approach for this tumor. However, the antigenic repertoire expressed by this tumor is still poorly defined [6]. Devising an efficient strategy to induce and expand *in vitro* T cells specific for the autologous tumor would be critical to implement both antigen-discovery and active or adoptive immunotherapy approaches in NSCLC.

The mixed lymphocytes tumor cultures (MLTC) have been utilized as effective means to induce high numbers of T cells specific for autologous melanoma [7], [8], [9], [10], [11], [12]; however, their application to other types of tumor has proved less favorable, possibly owing to the reduced antigenicity and/or immunogenicity of non-melanoma cancers. More recently, it became clear that a major pathway for antigen presentation *in vivo* relies on the indirect uptake of antigens, whether cell-associated or not, by Dendritic Cells (DCs), the most powerful antigen presenting cells in the immune system. This indirect antigen presentation pathway, named “cross-presentation”, can be successfully reproduced *in vitro* owing to the possibility to grow DCs *ex vivo*, and works efficiently with cell-associated, tumor-derived or viral antigens [13], [14], [15], [16], [17].

T cell specificity is exclusively determined by the T cell receptor (TCR), therefore the transfer into recipient polyclonal T cells of TCR genes, isolated from a high affinity tumor-specific T cell clone, generates T cell lines carrying the original clonotypic tumor-specificity. TCR-grafted T cells have been demonstrated to be functionally active both *in vitro* [18] and in *vivo* [19], [20]. Recently, the first TCR gene therapy trial in patients with melanoma demonstrated the clinical feasibility of the approach [21]. Due to the competition for cell surface expression with the endogenous molecules and the potential formation of mixed TCRs, the expression level of the transduced TCR is frequently reduced in comparison with the endogenous one, resulting in a poor functional activity of engineered T cells [22]. Among the strategies that have been implemented in order to maximize the likelihood of the intramolecular pairing of grafted TCR α and βchains in tranduced human T cells, a relatively straightforward one utilizes chimeric human-mouse TCR α and βchains [23], [24]. The chimeric TCRs consisting of a human variable and a mouse constant region, in fact, disfavors the mispairing between the transduced and the endogenous TCRchains, markedly increasing the correct expression of the grafted TCR αβheterodimers [23].

In this study, we have systematically investigated strategies to generate, propagate and expand CTLs specific for autologous NSCLC from PBMCs, a convenient source of lymphocytes, considering that it is not always possible to obtain tumor-infiltrating lymphocytes (TILs) from patients. We demonstrate that *in vitro* cross-presentation of NSCLC-derived tumor antigens by autologous DCs is exceedingly more efficient than the classic MLTC for the induction of tumor-specific CTLs. Furthermore, we show that it is possible to overcome the poor *in vitro* growth and expansion capacity of NSCLC-specific CTLs by grafting, via lentivirus-mediated transduction, their human V-mouse C chimerc TCR α and β chain genes into primary allogeneic T cells. This generates CD4^+^ or CD8^+^ T cell lines that stably express the grafted TCR clonotype and can be easily expanded to substantial numbers *in vitro* by standard culture conditions. Expression of the chimeric TCR redirects recognition of the transduced allogeneic T cells against the autologous NSCLC.

## Results

### MLTCs fail to induce NSCLC-specific CD8^+^ T cells

To determine the best experimental approach to generate CD8^+^ T cells specific for autologous NSCLC, a tumor cell line of the large cell carcinoma subtype was established from the surgical specimen of the CaPo13 NSCLC patient. CaPo13 cells expressed HLA-B and C but not HLA-A isotypes, as detected by FACS analysis (data not shown). To improve its immunogenicity, the cell line was transduced with the costimulatory molecules CD80 (B7-1), which was efficiently expressed on the cell membrane (data not shown).

**Figure 1:**
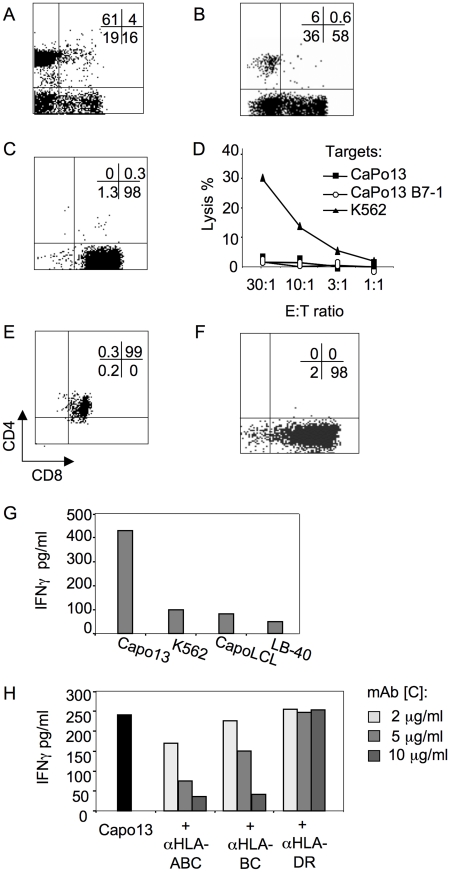
MLTCs fail to induce NSCLC-specific T cells. PBMCs from CaPo13 patients were cultured *in vitro* with irradiated autologous NSCLC cells engineered with CD80/B7-1. T cell line phenotype A) at the time of induction and B) after one week of MLTC; C) CD8^+^ enrichment by immunomagnetic sorting; D) induction of DP cells after the fourth restimulation; E) citotoxicity test performed on the specific targets CaPo13± B7.1 and on the NK target K562; F) CD8 expression by the single NSCLC-specific TCC 22N obtained by cloning the T cell line depicted in C; G) IFNγ production by TCC 22N after co-culture with the specific target CaPo13 cells, the NK cell K562 target, the autologous CapoLCL cell line, the unrelated melanoma cell line LB-40; H) TCC 22N recognition of CaPo13± the indicated HLA-blocking mAbs. Panels A–F show one representative experiment of three independent MLCTs performed. Panels G–H show one representative experiment of two performed.

CaPo13-B7-1 cells were used to stimulate autologous PBMCs according to established MLTC protocols. The frequency of CD8^+^ T cells increased from the initial 16% (Figure 1A) up to 58% of the cells in culture (Figure 1B) after one week of culture in the presence of irradiated CaPo13-B7-1 cells, IL-2 and IL-7. The CD8^+^ T cells present in the culture were enriched by positive immunomagnetic sorting, resulting in a population of 98% CD8^+^ T cells (Figure 1C). This polyclonal CD8^+^ T cell line did not kill the autologous CaPo13 tumor cell lines in a standard killing assay (Figure 1E). A weekly restimulation regimen with irradiated CaPo13-B7-1 cells and cytokines did not expand T cells able to kill the autologous tumor cells. Instead, three or four rounds of restimulation lead to a progressive replacement of the CD8^+^ T cells with CD4^+^CD8^+^ DP ones (Figure 1D), endowed with antigen non-specific killing activity (data not shown). This result was consistently obtained in two subsequent MLTC attempts.

After cloning the CD8^+^ T cells at the time of CD8-immunomagnetic enrichment, we could identify only one T cell clone (TCC), out of 500 tested, which specifically recognized autologous NSCLC cells. TCC 22N expressed CD8 (Figure 1F) and produced IFNγ following the specific recognition of CaPo13 cells in a MHC class I restricted manner (Figure 1G–H).

An independent set of MLTCs performed with a NSCLC cell line established from the tumor of a different patient resulted in the induction of a tumor non-specific DP T cell population, expanded from the patient's PBMCs (data not shown).

Collectively, this set of data suggested that the MLTC approach is unsuitable to efficiently induce CTLs specific for the autologous tumor from PBMCs in the NSCLC setting.

### Efficient induction of NSCLC-specific CD8^+^ T cells by DC-mediated cross-presentation of autologous irradiated tumor cells

**Figure 2:**
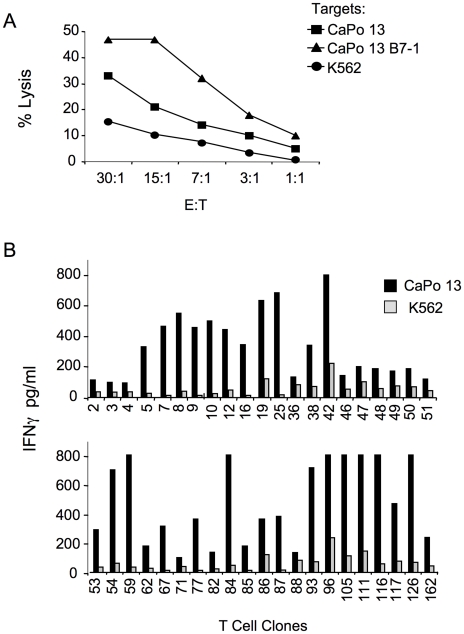
CD8^+^ T cell lines induced *in vitro* by DC-mediated cross-presentation specifically kill autologous CaPo13 cells. Immunomagnetically purified CD8^+^T cells were cultured with autologous DCs, both irradiated CaPo13 lung cancer cells and CD4^+^ T cells, hrIL-7 and hrIL-12. A) Citotoxicity assay performed after two rounds of stimulation on the specific target CaPo13 cells CaPo13± B7.1 and on the NK cell target K562; B) TCCs obtained by limiting dilution were co-cultured overnight with the specific target CaPo13 (black bars) or the NK cell target K562 (grey bar). IFNγ secretion was determined by ELISA. Clones were considered specific for the autologous NSCLC cells CaPo13 if their IFNγ production was at least three times higher than the one induced upon recognition of the NK target. The graph shows the results of the rapid screening with the first 42 TCCs obtained.

We next evaluated the capacity of inducing anti-tumor CTLs *in vitro* by cross-presentation of irradiated CaPo13 cells using autologous DCs and purified autologous T cells. After two rounds of weekly restimulations, the culture contained 70% of CD8^+^ T cells (data not shown) that displayed a specific and vigorous killing against the autologous tumor cell lines (Figure 2A).

**Figure 3:**
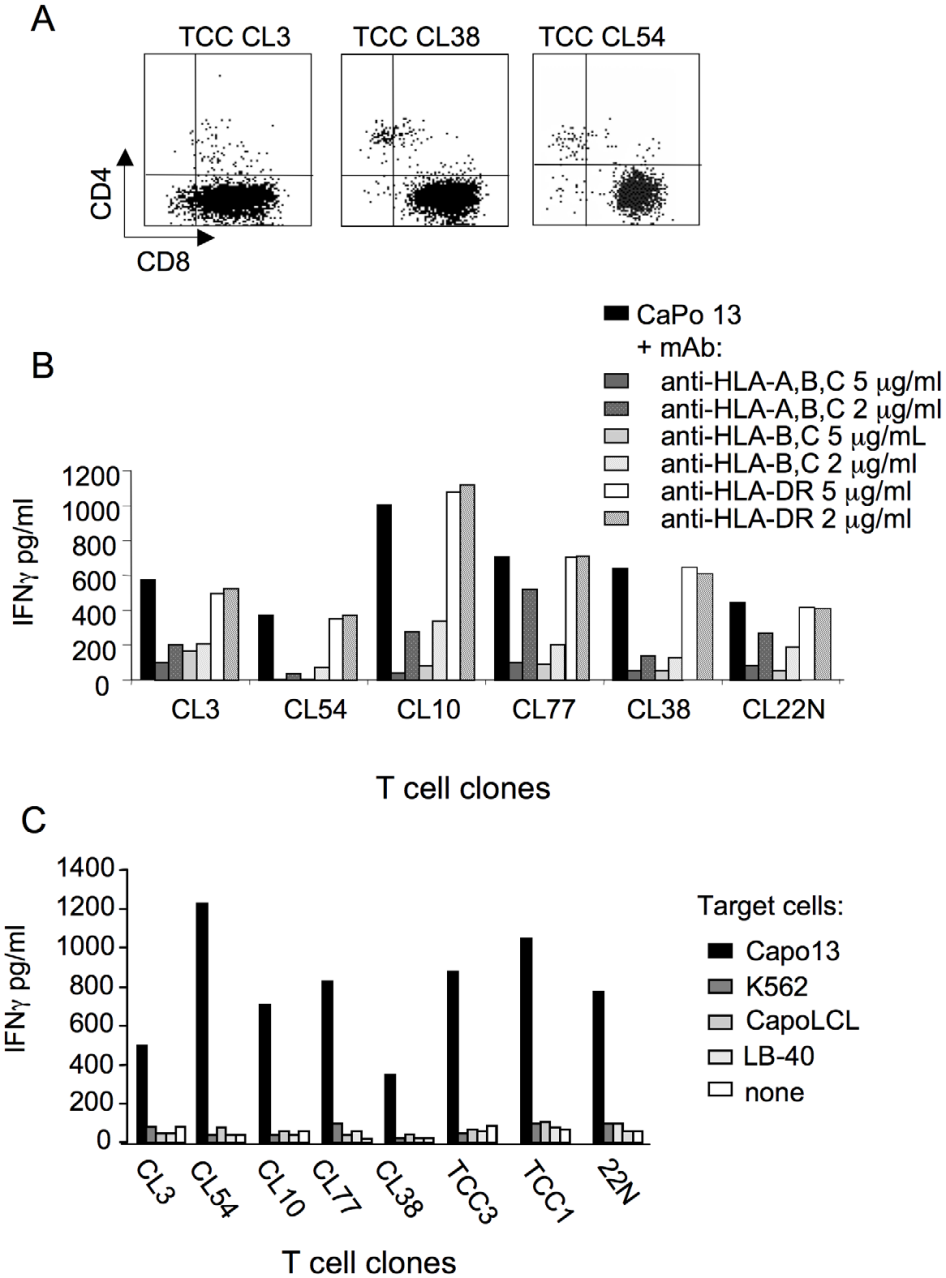
Characterization of the NSCLC-specific CD8^+^ TCC obtained by *in vitro* cross-presentation. TCCs responding to CaPo13 and not to K562 cells upon rapid screening were retested against a variety of targets to confirm their specificity. A) CD8 coreceptor expression of representative TCCs; B) TCCs recognition of CaPo13 cells in the presence or not of antibodies blocking HLA molecules; C) IFNγ production by selected TCCs after overnight co-culture with the specific target CaPo13, the NK specific target K562, the autologous cell line CapoLCL and the unrelated melanoma cell line LB-40. Representative clones are shown. Panel B-C show one representative experiment of two performed.

The high frequency of tumor-specific CD8^+^ T cells contained in the cell line was confirmed by the rapid screening of the TCCs obtained by limiting dilution: 52 out of 184 tested clones produced IFNγin response to autologous CaPo13 and not to K562 cells (Figure 2B and data not shown). TCCs specific for CaPo13 cells expressed the CD8 coreceptor (Figure 3A) and recognized the autologous lung cancer cell line in a HLA class I restricted manner, but not antigen-unrelated target cells, including the autologous Capo13 LCL line and the allogeneic, HLA-unrelated melanoma cell line LB-40 (Figure 3B and 3C).

Together, these data suggested that DC cross-presentation of tumor-derived antigens to autologous T cells *in vitro* is an efficient strategy to induce and expand tumor-specific CD8^+^ T cells also in the NSCLC setting.

### Limited TCR clonal heterogeneity of the T cell clones specific for the autologous CaPo13 NSCLC

To investigate the clonal composition of the NSCLC-specific T cell response induced *in vitro* by DC-mediated cross-presentation of tumor-associated antigens, the TCRVβ and Vα repertoire of 10 selected CaPo13-specific TCCs was investigated by RT-PCR using a panel of oligonucleotide primers specific for 29 Vα and 24 Vβ families respectively. These clones were selected for exhibiting a consistently greater response to CaPo13 cells. The analysis showed the presence of sister clones, sharing identical TCRs (Table 1) as confirmed by sequencing analysis. Globally, we identified four different TCR clonotypes expressed by the CaPo13-specific CD8^+^ T cell clones induced by DC cross-presentation plus one T cell clonotype induced by MLTC, suggesting the expansion *in vitro* of a limited number of T cell precursors specific for the autologous NSCLC.

**Table 1:** The TCR repertoire of anti-CaPo13 T cell clones displays a limited diversity.

TCC	TCR	Vα	Jα	Vβ	Jβ	Cβ	Clonotype
**22N**		13.1	27	3	1.1	1	I
**CL3**		12	40	13.1	2.1	2	II
**CL8**		12	40	13.1	2.1	2	II
**CL38**		12	40	13.1	2.1	2	II
**CL54**		20	15	5.2	1.6	1	III
**CL10**		20	15	5.2	1.6	1	III
**CL85**		20	15	5.2	1.6	1	III
**CL77**		7	37	7	2.7	2	IV
**TCC1**		7	37	7	2.7	2	IV
**TCC3**		1	31	6	2.7	2	V

T cell clones with the same TCR α and β chains are grouped in the same clonotype. The first TCR clonotype listed, CL22N, represents the only tumor-specific TCC obtained by MLTC.

### TCRs expressed by the tumor-specific T cell clones isolated *in vitro* are detected in TILs

**Figure 4:**
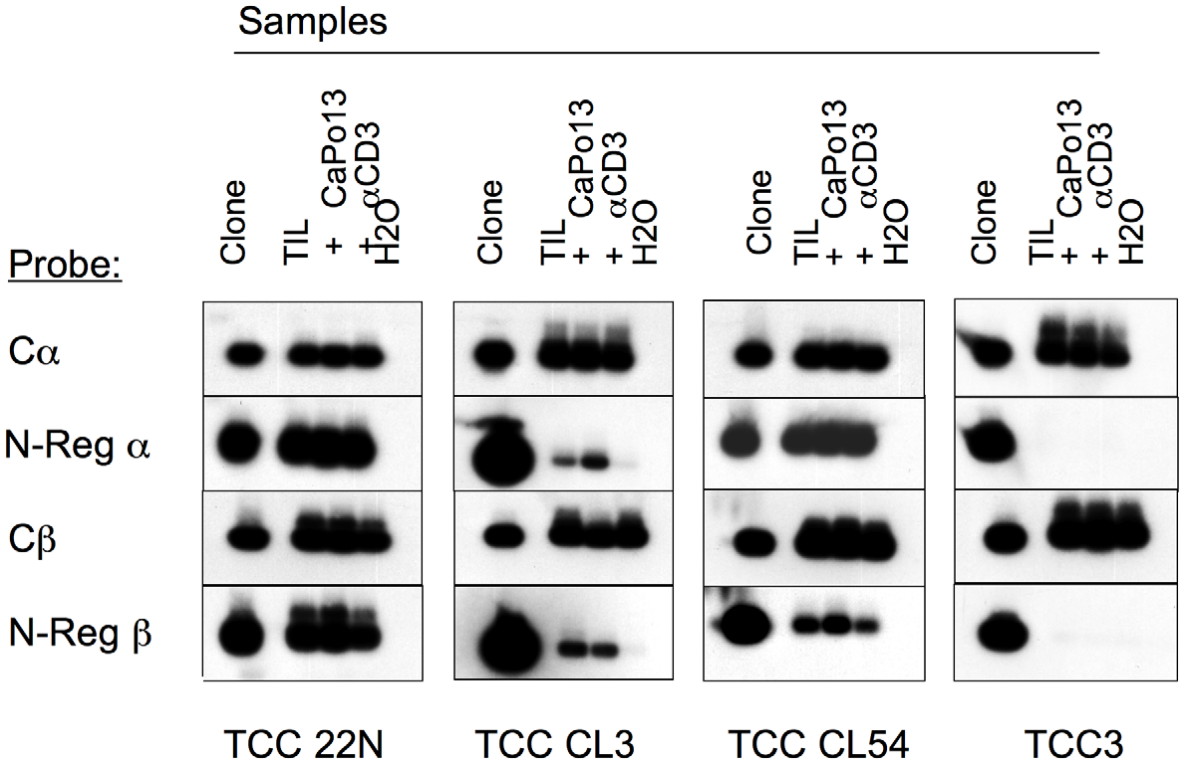
TCR molecular fingerprints of the selected NSCLC-specific TCCs in TILs. The presence of the NSCLC-specific TCR clonotypes isolated *in vitro* was determined by PCR-oligotyping in TILs of the CaPo13 patient. PCR oligotyping was performed for both TCR α and β chains. A probe specific for the constant region (C), which therefore hybridizes with any PCR product, is used as control. The agarose gel was blotted onto nylon filters, and hybridized sequentially with a probe specific for the TCR α and β N-regions and for the TCR C α and β regions. Four TCC clones are shown.

To determine whether the NSCLC-specific CD8^+^ T cell clones isolated *in vitro* might have played any role in the spontaneous anti-tumor response *in vivo,* we investigated whether the molecular fingerprints of the NSCLC-specific TCR clonotypes identified in this study were present at the tumor site. Mononuclear cells infiltrating the CaPo13 tumor specimen were thawed and part of them immediately processed for RNA extraction (primary TILs), while a part was expanded *in vitro* by culturing for 10 days in the presence of IL-2 (20 IU/ml) and either irradiated CaPo13 cells or anti-CD3 mAb. The TCR Vα and Vβ chains of three out of five TCR clonotypes analyzed were detected in TILs (TCC 22N, TCC CL3, TCC CL54). The molecular fingerprints of TCC3 (Figure 4) and TCC1 (not shown) could not be detected in any experimental condition. TCC CL3 TCR fingerprint was undetectable in the TIL sample expanded by anti-CD3 mAb stimulation, as compared to the sample obtained *ex vivo* or expanded *in vitro* by CaPo13 cells. This suggested that TCC CL3 could be outcompeted *in vitro* by the growth of other TCCs present in TILs that responded better to the polyclonal stimulus. Indirectly, this result also confirmed that TCC CL3 was specific for the autologous tumor cells. The presence of three out of five clonotypes among TILs was confirmed by heteroduplex analysis (data not shown).

Altogether, these findings suggested that the NSCLC-specific CD8^+^ TCCs isolated *in vitro* participated in the immune surveillance of the autologous tumor *in vivo*.

### Propagation and expansion of CaPo13-specific TCC CL3 TCR clonoype by TCR transfer into primary allogeneic T cells

**Figure 5:**
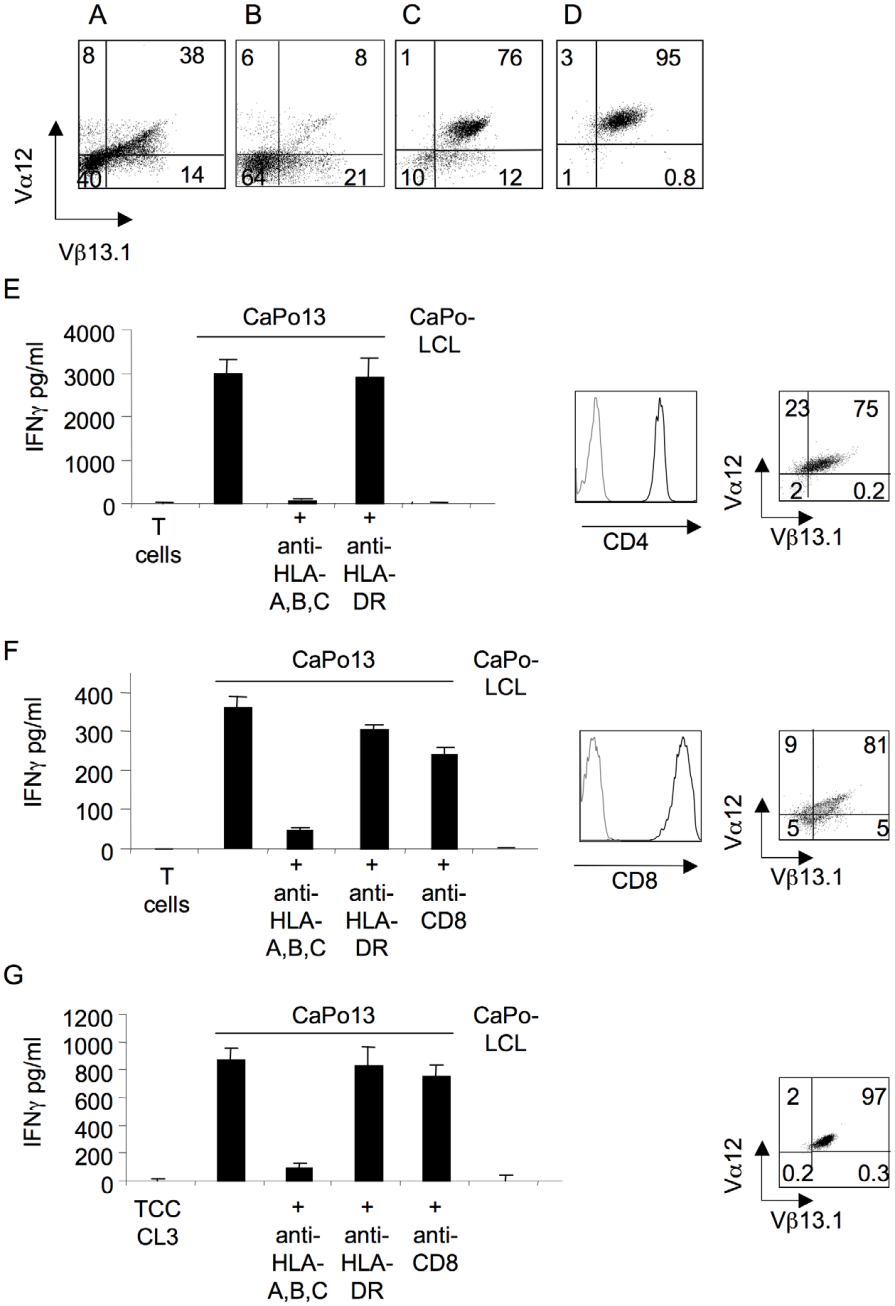
Selection of TCC CL3 TCR transduced allogeneic T cells specific for CaPo13 cells. Allogeneic T cells were transduced two days after activation with a lentiviral vector encoding the chimeric TCC CL3 Vα12-Jα40-mouse Cα and Vβ13.1-Jβ2.1-mouse Cβ TCR chains. Expression of the transferred TCR was detected on transduced T cells: A) at day 5 from activation; B) at day 12 from activation; C) after one cell sorting with anti-mouse TCRβ mAb and two rounds of stimulation with irradiated CaPo13 cells; D) after two rounds of polyclonal stimulation following the last stimulation with irradiated CaPo13 cells. Ag-specific response and TCR expression of E) cell sorted CL3 TCR^+^ CD4^+^ cell line; F) cell sorted CL3 TCR^+^ CD8^+^ cell line and G) the original TCC CL3 cultured with the autologous NSCLC CaPo13 cells ± anti-HLA-I, anti-HLA-DR or anti-CD8 blocking mAbs. IFNγ production was determined by standard ELISA. Cells were stained with anti-CD3, anti-Vα12, anti-Vβ13.1, anti-CD8 or anti-CD4 mAbs. One representative experiment of three performed is shown.

To overcome the technical hurdle represented by the poor expansion of these TCCs *in vitro* by standard culture conditions, we sought to transfer the CaPo13-specific TCR clonotypes into fast growing primary allogeneic T cells. We generated chimeric TCR molecule containing the human V region linked to the murine C region, with the threefold purpose to: *i* enhance the correct TCR α/β pairing of the exogenous chains; *ii* disfavoring mis-pairing with the endogenous TCR α and β chains; and *iii* improve the assembly with the human CD3 complex [23], [25]. Moreover, the presence of the mouse Cβ region expressed by the chimeric TCR would also permit its specific detection in human T cells, thanks to a mAb specific for the mouse TCR Cβ. As a proof of concept, we sought to transfer the TCR expressed by TCC CL3 because it expressed the Vα 12 and Vβ 13.1 gene segments that are recognized by specific mAbs, facilitating their tracking upon transduction into allogeneic T cells. Futhermore, the molecular fingerprint of this TCR was detected in the TILs of CaPo13 patient. The chimeric TCR α and β chain of TCC CL3 were cloned as a single transcriptional cassette under the control of a common SFFV (spleen focus forming virus) promoter into the pHRSIN-Bx-IRES-Em lentiviral vector. As shown in Figure 5A, 72 hours after transduction (day +5), 38% of policlonally activated T cells co-expressed the transduced TCR Vα12/Vβ13.1. This frequency was markedly increased in comparison to that of the same TCR V regions pairing in non-transduced T cells (0,7%, data not shown), indicating that the bicistronic lentiviral vector efficiently transferred the chimeric TCR ~αβ into PBMCs. The frequency of cells expressing the transduced chimeric TCR CL3, however, decreased markedly already by day 12 after transduction (Figure 5B) and reached background levels (i.e. untransduced T cells) by day 15. Cell sorting for the expression of the TCR CL3-transduced T cells followed by a polyclonal stimulation with anti-CD3 mAb, allogeneic irradiated PBMC and hrIL-2 did not rescue the surface detection of the chimeric TCR (data not shown). However, two to three weekly antigen-specific restimulations of the TCR CL3-transduced T cells with irradiated CaPo13 cells, coupled with a round of cell sorting of T cells expressing the transduced chimeric TCR Vα12/Vb13.1, resulted in a population of T lymphocytes that displayed a nearly homogenous expression of the transduced TCR over time (Figure 5C). Once established by this protocol, the TCR CL3-transduced T cell lines could be maintained and expanded by polyclonal stimulations, without losing the expression of the transduced TCR (Figure 5D).

### Allogeneic CD4^+^ or CD8^+^ T cell stably transduced with a NSCLC-specific chimeric TCR recognize CaPo13 tumor cells

We next sorted for CD4 and CD8 expression the T cells homogenously and stably expressing the transduced TCR CL3 and tested them for the recognition of CaPo13 cells. As shown in Figure 5 E–F, both CD4^+^ and CD8^+^ TCR CL3 transduced T cells (CL3 TCR^+^CD4^+^ and CL3 TCR^+^CD8^+^) produced IFNγ in a HLA class I-restricted manner upon recognition of CaPo13 cells. Production of IFNγ upon recognition of CaPo13 cells by both the original TCC CL3 and the CL3 TCR^+^CD8^+^ T cells occurred also in the presence of anti-CD8 blocking mAb (figure 5 F–G). This demonstrated the coreceptor independence of CL3 TCR and it was consistent with the recognition of CaPo13 cells also by the CL3 TCR^+^CD4^+^ transduced T cells. Although CL3 TCR^+^CD4^+^ cells efficiently produced IFNγin response to an Ag-specific stimulation, they produced negligible quantities of IL-2 (mean 6.5 pg/ml; unstimulated control T cells <1 pg/ml), suggesting that these cells exhibited essentially an effector-memory phenotype owing to the recurrent stimulations *in vitro* in the presence of IL-2. A different stimulation protocol *in vitro*, based on the use of IL-7 amd IL-15 in lieu of IL-2, plus anti-CD28 costimulation, might be required in order to expand TCR-tranduced T cells endowed with IL-2 production [26].

**Figure 6:**
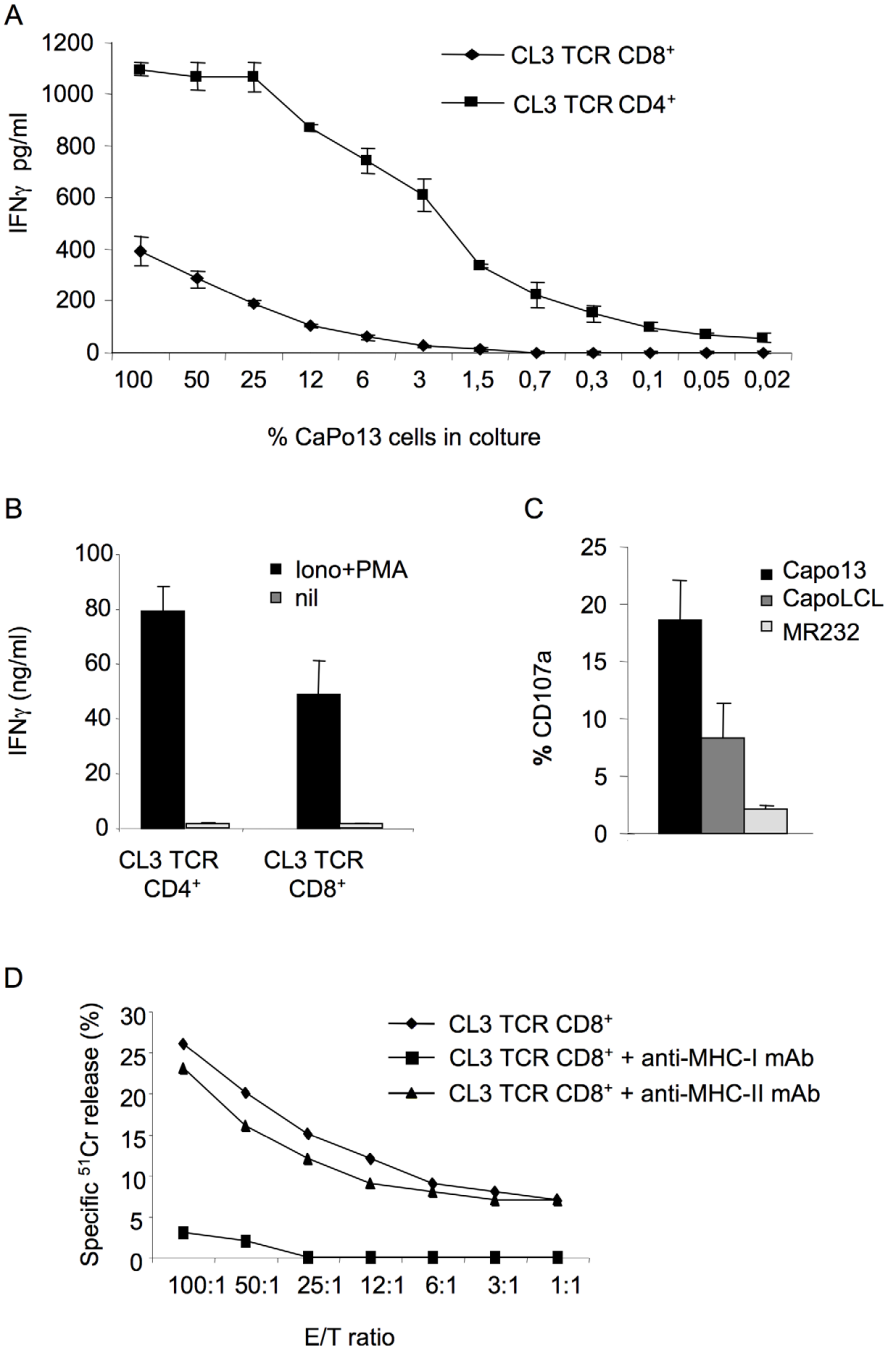
TCR-transduced CD4^+^ and CD8^+^ T cells have different Ag responsiveness. The tumor-specificity, responsiveness and cytolytic potential of the CL3 TCR^+^ CD4^+^ and CL3 TCR^+^ CD8^+^ T cell lines were investigated. A) CL3 TCR CD4^+^ and CD8^+^ T cells were cultured with CaPo 13 cells diluted into COS7 cells at different percentages; B) CL3 TCR CD4^+^ and CD8^+^ T cells were activated or not with PMA and Ionomycin. After 48 h, IFNγ production was determined by standard ELISA. C) CL3 TCR CD8^+^ cells were cultured with the specific target CaPo13, the unrelated NSCLC cell line MR232 and the autologous cell line CapoLCL for 5 hours and then analysed for CD107a expression. One representative experiment of two performed is shown. D) CL3 TCR CD8^+^ cells were cultured with ^51^Cr-labelled CaPo13 for 4 h with or without the addition of mAbs specific for either MHC class I (HLA-A, B, C) or MHC class II (HLA-DR), and killing was determined as described [15]. One representative experiment of two performed is shown.

The sensitivity of transduced CD4^+^ and CD8^+^ T cell lines for the specific target were directly compared by co-culturing them with CaPo13 cells diluted at different percentages into irrelevant COS7 cells. CL3TCR^+^CD4^+^ cells displayed an 8 times higher Ag-specific responsiveness than the CL3 TCR^+^CD8^+^ ones and were still able to produce IFNγwhen CaPo13 cells were diluted 1∶500 (Figure 6A). This could be explained by the greater intrinsic capacity of the CD4^+^ subset to produce IFNγ upon activation, as suggested by the different response of the two CL3TCR transduced T cell subsets to polyclonal activation (Figure 6B). However, only CL3 TCR^+^CD8^+^ cells displayed tumor-specific citolytic potential, as suggested by the up-regulated CD107a upon co-culture with CaPo13 cells in these cells and not in the CL3TCR^+^CD4^+^ ones (Figure 6C and data not shown). CL3 TCR^+^CD8^+^ cells killed in a MHC class I-restricted manner CaPo 13 cells also in classic 4 h ^51^Cr-release assays (Figure 6D), confirming that the CL3 TCR transfer endowed these allogeneic T cells with actual NSCLC-specific killer effector functions.

## Discussion

In this study we explored ways of generating CTLs directed against NSCLC from autologous PBMCs. We showed that conventional MLTCs, in which PBMCs are co-cultured with an autologous NSCLC cell line, was inefficient in inducing tumor-specific T cells. Moreover, MLTCs resulted in the induction of T cell lines displaying CD4^+^CD8^+^ DP phenotypes and non-specific killing activity. It is unclear why direct presentation of tumor-antigens to autologous T cells by NSCLC cells should result in the expansion of antigen non-specific DP T lymphocytes. These cells derive from purified CD8^+^ T lymphocytes (homogenously negative for CD4 expression) stimulated three-four times in MLTCs with the autologous tumor cells. It is possible that these cells derive from CD8^+^ T cells that have upregulated the CD4 coreceptor generating DP T cells endowed with antigen non-specific effector functions. CD8^+^ T cells were indeed shown to upregulate CD4 after activation [27]. Alternatively, the antigen non-specific DP T lymphocytes cells could represent a pre-formed population, which is present at very low frequency at the beginning of the culture and progressively expanded upon interaction with autologous lung cancer cells. DP T cells endowed with non-specific killing activity were obtained by MLTC with renal cancer cells [28], suggesting that MLTC with tumor cells other then melanoma might often result in the induction of this kind of T cells.

By the combined use *in vitro* of DCs, irradiated CD4^+^ T helper cells, IL-12 and IL-7, we have demonstrated that cross-presentation of dying tumor cells resulted in the generation of a higher number of tumor specific TCCs, compared to any other published attempt to induce NSCLC specific CTL clones by MLTCs. We have elaborated this *in vitro* culture system from the notion that optimal induction of CD8^+^ T cells specific for exogenous antigens *in vivo* requires the combination of DC dependent cross presentation, help by CD4^+^ T cells and the addition of IL-12 and IL-7, whose presence was shown to be critical for the efficient induction of anti-tumor effector T cells by Montagna et al [15]. IL-12 and IL-7 were utilized in the induction phase of the tumor-specific T cell response *in vitro*, because of the known function of IL-12 in directing the acquisition of strongly anti-tumor Th1/Tc1 effector functions, and of IL-7 in supporting the growth of human CD8^+^ T cells [29], [30], [31].

Despite the large number of TCCs obtained by DC-mediated *in vitro* cross-presentation, the diversity of the TCR repertoire displayed by these cells clones was restricted to four different TCR clonotypes. This does not seem to be an anomaly in the CD8^+^ T cell response. In general, the clonal heterogeneity of CD8^+^ T cell response specific for exogenous (microbial or viral) antigens is quite limited, yet it is sufficient to control infections [32], [33]. Furthermore, in melanoma patients, the CD8^+^ T cell responses specific for immunodominant epitopes derived from the self shared tumor antigens MelanA/Mrt1 or NY-ESO1 display also a limited clonal heterogeneity. Therefore, the little clonal diversity exhibited by the NSCLC-specific CD8^+^ T cell clones that we have isolated might reflect the intrinsic physiological property of the CTL response, and this TCR clonality could be large enough to support the potential recognition of different antigenic epitopes expressed by NSCLC cells.

The NSCLC-specific T cell clonotypes that we have isolated *in vitro* may be representative of the spontaneous anti-tumor response elicited *in vivo* by the presentation of TAA to the patient's T lymphocytes. In support of this, we have detected the TCR fingerprints of three out of five of these clonotypes in the TILs isolated from the autologous tumor, suggesting that these three TCR clonotypes may be involved in the spontaneous anti-tumor response. The two clonotypes that were not found could either not be present or present below the detection limits of the PCR-oligotyping in the tumor site at the time of surgery. Alternatively, it is possible that these two TCR clonotypes were primed *in vitro* against the autologous lung cancer by DCs.

We showed that it is possible to overcome the difficulties to maintain in culture CTLs specific for self-antigens by transferring their TCR into allogeneic polyclonal T cells, which could be easily expanded in culture. Tracking the expression of the transferred TCR is a critical issue to determine the frequency of transduced T cells and the degree of mispairing between the transferred and the endogenous TCR α and β chains. This can be done by using mAbs specific for the Vα and Vβ regions of the transduced TCRs or by the use of multimers of the cognate MHC-peptide complex recognized by the transduced TCR. However, TCR V region-specific or MHC-peptide tetramers are not always available. The use of chimeric TCR α and β chains with human V and mouse C domains might, at least in part, overcome these problems not only by increasing the amount of exogenous homologous TCRαβ pairing [23], [24], but also by enabling the tracking of the grafted TCR molecules in transduced human T cells by using mAbs specific for the mouse Cβ region, regardless the availability of TCR Vα and/or Vβ specific mAbs.

We successfully transduced the CL3 TCR chimeric α and β chains in around 20–40% of peripheral blood derived human T cells in all performed infections (data not shown), as detected three days after infection. We constantly observed a decrease in the frequency of T cells expressing the transduced TCR already by day +5 post-infection. Furthermore we observed the same decrease after polyclonal restimulation of the transduced CL3 TCR^+^ T cells enriched by FACS sorting. We do not have at present a ready explanation for the rapid loss of the grafted TCR surface expression in transduced primary human T lymphocytes. A simple explanation could be the lack of transgene integration in the majority of transduced T cells. Infecting primary T cells at the same MOI with the control vector carrying the GFP as transgene, however, resulted in a stable percentage of GFP expressing T cells over time (data not shown). On the other hand, the transduction of the lentiviral vector carrying the CL3 TCRα and β chain into human primary T cells did not result in a proliferative disadvantage or increased cell death compared to the untransduced T cells (G. Canderan, data not shown).

However, consistent with published data [34], cyclic antigen-specific stimulations successfully stabilized the expression of the transferred CL3 TCR in the transduced T cells. It is possible that the progressive stabilization of the transferred CL3 TCR expression on the membrane of the transduced T cells might be due to a selection in culture of a limited set of T cell clones whose endogenous TCR is permissive for the expression of the exogenous one. Future analysis of the clonal composition of long-lasting T cell lines expressing the transduced CL 3 TCR will contribute to address this issue.

We showed that, in addition to CD8^+^, also CD4^+^ T cells expressing the transduced TCR CL3 efficiently recognized CaPo13 cells. This is consistent with the CD8-independence of the original TCC CL3 clone. Actually, the CL3 TCR^+^CD4^+^ T cells display a somewhat higher responsiveness against CaPo13 target cells than the CL3 TCR^+^CD8^+^, based on their IFNγ production, reflecting their higher intrinsic efficacy as inflammatory cells. This aspect may be interesting in the perspective of a clinical application of TCR transfer in selected CD4^+^ or CD8^+^ T cells for adoptive immunotherapy of cancer. The use of CD8 co-receptor independent tumor-specific TCRs would permit to exploit both CD4^+^ and CD8^+^ T-specific effector functions.

In conclusion, this study defines a strategy to efficiently induce and propagate *in vitro* high numbers of T cells carrying a TCR specific for autologous NSCLC. This strategy may be easily adapted to all form of human malignancies. Furthermore, given that the T cell clones isolated are representative of an anti-tumor response spontaneously aroused in the CaPo13 patient, the identification of the antigens recognized by them might give interesting clues on the NSCLC/host immune system interactions.

## Materials and Methods

### Ethics Statement

The publication of the data obtained in this study with the lung cancer cell line CaPo13 has been authorized by the Institutional Ethical Committee of San Raffaele Scientific Institute (#08-04-2010) in the light of the consideration that this cell line was established from the surgical specimen of a patient before 2002, when the approval of the Institutional Ethical Committee for such procedures for research purposes became formally required by national law.

### Cell cultures

CaPo13 and MR232 NSCLC cell lines, LB-40 melanoma cell line (kindly obtained from Ludwig Institute of Cancer research, Bruxelles), K562 cell line (ATCC CCL 243), transduced T cell lines and LCL cell lines were kept in complete RPMI 1640 medium containing 10 U/ml penicillin and streptomycin, 2 mM L-glutamine, 1 mM sodium pyruvate, 1% non-essential amino acids (*Gibco*) supplemented with 10% heat-inactivated FCS (*Euroclone*) (RPMI-FCS). T cell clones (TCCs) were grown in complete RPMI medium supplemented with 5% human serum (RPMI-HS) and cytokines as indicated in text. 293T cells were cultured in IMDM (*Gibco*) containing 10 U/ml penicillin and streptomycin, 2 mM L-glutamine and 10% heat-inactivated FCS.

### Establishment of CaPo13 NSCLC cell lines

Single cell suspension was prepared by digesting finely chopped tumor sample with 1 mg/ml collagenase, 0,1 mg/ml hyaluronidase and 0,02 mg/ml DNAse (*Sigma*) at 37C° for 4 h. Tumor samples were obtained from the routine pathological analysis of the surgical specimens. Tumor cells were separated from infiltrating lymphocytes on a 1.077-1.055 discontinuous Ficoll gradient and plated in flasks in complete RPMI-1640 medium and D-Valine to inhibit fibroblasts outgrowth [35]. Tumor-infiltrating lymphocytes (TILs) were collected and frozen. Early-passage tumor cell lines were used to minimize possible modifications of primary characteristics as a consequence of extensive in vitro culturing. B-lymphoblastoid cell lines (Capo-LCL) were established from the patient cryopreserved PBMCs by the incubation with EBV-containing supernatant from the B95.8 cell line (ATCC). CaPo13 were transduced with the retroviral vector LXSDN [36] carrying the reporter gene NGFR and the CD80 cDNA. Cells expressing NGFR were isolated by immunomagnetic sorting (*Dynabeads*).

### Induction of anti-CaPo13 T cell lines by autologous MLTCs

CD80-tranduced CaPo13 cells (CaPo13-B7-1) were irradiated at 100 Gy and plated with autologous PBMCs at the ratio of 3×10^4^ NSCLC cells for 1,5×10^6^ PBMCs in complete RPMI-HS medium. After 48 h 10 IU/ml hrIL-2 (*Roche*) and 5 ng/ml hrIL-7 (*R&D Systems*) were added.

### Cross-priming of tumor-specific T cells *in vitro*

CaPo13-specific CTL-lines were generated by a modification of a published protocol [15]. Autologous dendritic cells were obtained from PBMC according to published protocols [15]. CD8^+^ T cells were isolated from PBMCs by positive immunomagnetic sorting (*Miltenyi Biotec*). Complete RPMI-HS medium was further supplemented with 10 ng/ml IL-7 (*R&D Systems*) and 10 pg/ml IL-12 (*R&D Systems*). For induction of anti-tumor CTL-lines, patient's CD8-enriched PBMCs (0.5 to 1×10^6^ cells/ml) were added to 48-well plates and co-cultured with irradiated (100 Gy) autologous CaPo13 cells (5×10^5^ cells/ml), irradiated (60 Gy) autologous CD8-deprived PBMCs (3 to 5×10^5^ cells/ml) and autologous DCs (2×10^5^ cells/ml) in a final volume of 1 ml. After 7 to 10 days, cultures were restimulated with irradiated (100 Gy) CaPo13 (5×10^5^ cells/ml) and irradiated (60 Gy) autologous PBMCs. Two days later, 25 IU/ml of hrIL-2 were added to the cultures. The same protocol was used for each successive round of stimulation.

### ^51^Cr-release assay

Cytotoxic activity of CTL-lines was tested in an 4 hr ^51^CR-release assay as described [37], using an effector/target (E/T) ratio that ranged between 30∶1 and 5∶1. Results are expressed in lysis percentage for each E/T ratio using the sequent formula Lysis %  =  (CPM-CPM spontaneous release)/(CPM total release-CPM spontaneous release) ×100.

### Isolation and expansion of T-cell clones specific for the autologous CaPo13 NSCLC cells

Polyclonal CTL lines recovered after two rounds of CaPo13-specific stimulation were cloned as described [38]. After 12 to 14 days of culture, all growing wells were harvested and expanded in the presence of 100 IU/ml IL-2, phytohemagglutinin (PHA, 1 µg/mL), and allogeneic irradiated feeder cells (2×10^6^ cells/ml). The TCCs obtained were screened for IFNγ secretion in response to CaPo 13 cell stimulation by standard ELISA (*Pierce Endogen*). TCCs that reacted positively to CaPo13 were re-tested for their ability to recognize the specific target cell line CaPo13 and not the non-specific melanoma cell line LB-40 or the NK specific cell line K562. CTL clones (8×10^3^) were added to target cells (2×10^4^) in 96-well plates in complete RPMI-HS medium. Cell cultures were performed in the presence or absence of CaPo13 and different concentration of anti-HLA class I (W6/32) or anti-HLA-B-C (4E) mAbs. Supernatants were collected 48 hours later and the IFNγ concentration determined by standard ELISA.

### TCR repertoire analysis

Total RNA from CTLs, isolated with TRIZOL (*Invitrogen*), was converted into cDNA and PCR amplified using a panel of 29 forward TCR Vα specific and 24 Vβ specific oligonucleotide primers with reverse oligonucleotide primers complementary to the TCR C α or β region as described [39], [40].

### Oligotyping analysis on TILs

Frozen TILs were thawed and either immediately lysed for RNA extraction or expanded *in vitro* by CaPo13-specific stimulation (100 Gy irradiated CaPo13 cells) or by polyclonal stimulation (30 ng/mL OKT3 and 2×10^6^/ml 60 Gy irradiated feeders) in the presence of 100 IU/mL hrIL-2. Total RNA from the anti-CaPo13 TCCs and from the stimulated or unstimulated TILs was converted into cDNA and PCR amplified with oligonucleotide primers specific for the TCR V α and β segments expressed by each TCC and for TCR Cα and β regions (listed in [39], [40]). The PCR profile was: 10 min at 94°C followed by 30 s at 94°C, 30 s at 61°C and 30 s at 72°C for 35 cycles, and final 10 min at 72°C. The PCR products were run on a 1.5% agarose gel and blotted onto nylon membranes (*Hybond, Amersham*). Oligonucleotide probes specific for the TCR α and β chain N–regions (Table S1) of the 5 anti-CaPo13 TCCs were labelled and hybridized as described [40].

### Generation of lentiviral vector carrying the CL3 TCR α/β chimeric chains

The V-J TCRα and TCRβ gene segments of TCC CL3 TCR were amplified by PCR and cloned in frame with mTCR Cα and Cβ. The chimeric human Vβ13.1-mouse Cβ and human Vα12-mouse Cα were subcloned into the pIRES vector (*Clontech*). The entire Vβ-Cβ-IRES-Vα-Cα region was PCR amplified using a 5′ primer containing a Bcl2 restriction site (pIRES for 5′ AGTCTGATCACGACTCACTATAGGCTAGCCTCG; pIRESrev 5′ CCTCA CTAAAGGGAAGCGGC). The amplicon was digested with Bcl2 and NotI and cloned into the lentiviral vector pHRSIN-Bx-IRES-Em (kindly provided by Dr V. Cerundolo, Univ. of Oxford).

### Production of lentiviral vectors carrying the chimeric TCC CL3 TCR

Vector stocks were produced by Ca_2_PO_4_ transient transfection of 293T cells using a second-generation lentiviral vector system following published protocols. Breefly, subconfluent 293T cells in 15 cm dish were transfected with 12 µg, 16,25 µg and 6,25 µg of the pMD2.VSV-G, pCMVdR8.74 and pRSV-Rev packaging plasmid [41], [42] respectively (kindly provided by Dr Luigi Naldini, San Raffaele Scientific Institute), mixed with 32 µg transfer vector plasmid pHRSIN-CL3TCRαβ. Sixteen hours after transfection medium was replaced and virus was collected 30 hours after transfection. After filtering through a 0,22 mM filter (*Millipore*), the vector was purified and concentrated by ultracentrifugation at 20000 g. The average titer was evaluated on TCR deficient Jurkat 76 cells [24] by adding serial dilutions of concentrated vector to 2.5×10^5^ cells in a 48-well plate (*Nunc*). Cells were analyzed 96 hours after transduction for CL3 TCR expression by flow citometry. Transducing units were calculated using the sequent formula (number of cells in the well at the time of transduction) × (frequency of TCR positive cells) × (dilution factor)/volume of inoculum in mL  =  TU/mL.

### Transduction of allogeneic PBMCs by lentiviral vector carrying the chimeric TCC CL3 TCR

PBMCs were isolated from buffy coats by Ficoll-Hypaque gradient separation (*Pharmacia)*. T cells were activated with anti-CD3/CD28 immunomagnetic beads (*Dynabeads*) according to manufacturer's instructions and kept in RPMI-FCS complete medium supplemented with 5 ng/ml IL-15 and IL-7 (*R&D systems*). Two days after activation, 4×10^5^ T cells were infected in 500 µl complete medium at a multiplicity of infection (MOI) of 5 in a 48-well plate. Cells were infected overnight and then washed from the virus. After 72 h from infection, cells were stained with Vα12-FITC (Pierce *Endogen*) and Vβ13.1-PE (*Beckman coulter*) mAbs, fixed by paraformaldehyde (1%) and then analyzed by flowcytometry.

### Expansion and selection of TCC CL3 TCR transduced allogeneic T cells

Transduced allogeneic T cells (1×10^6^) were stimulated weekly with irradiated (100 Gy) CaPo13 (2×10^5^) and irradiated (100 Gy) RosiEBV LCL cells (2×10^4^) in 1 ml complete RPMI FCS medium supplemented with 100 IU/mL IL-2. Transduced cells underwent one or more cycles of cell sorting to enrich for TCC CL3 TCR expression by staining with mAb specific for mouse TCRβ (*BD biosciences*). Transduced T cells expressing either CD4 or CD8 coreceptor were obtained by cell sorting using co-receptor specific mAbs. Transduced T cells homogenously positive for TCC CL3 TCR surface expression (1×10^6^) were expanded by polyclonal stimulation (30 ng/mL OKT3, 25×10^6^ cells irradiated feeders (60 Gy), 5×10^6^ cells irradiated RosiEBV (100 Gy), 600 IU/mL rhIL-2) in 25 ml complete RPMI FCS medium.

### Activation *in vitro* of CD4^+^ and CD8^+^ T cells transduced with the chimeric TCC CL3 TCR

Specific T cell activation was determined by co-culturing CL3 TCR^+^ CD4^+^ or CL3 TCR^+^ CD8^+^ cells (10^4^) with target cells CaPo13 or CapoLCL (4×10^4^) in flat 96 wells for 48 hours. Blocking mAbs W6/32, L243 and OKT8 were added at 1.0 µg/mL. Direct comparison of CL3 TCR^+^ CD4^+^ and CL3 TCR^+^ CD8^+^ cells Ag responsiveness was performed by co-culturing effector T cells (10^4^) with CaPo13 diluted at different percentages in COS7 cells (total target cells 4×10^4^) for 48 hours. As control the two trasduced T cell lines were activated with 1 µg/ml Ionomycin and 50 ng/mL PMA. IFNγ production was determined by standard ELISA.

CL3 TCR^+^CD8^+^ T cell line killing activity was analyzed by CD107 mobilization or ^51^Cr release assay. T cells (2×10^4^) were co-cultured with CaPo13, CapoLCL or MR232 (2×10^4^) in the presence of 1 µl of CD107a-PE (*Ebioscience*) for 5 hours. The cells were washed with 0.02% azide and 0.5 mM EDTA, stained with anti-Vα12-FITC mAb (*Pierce Endogen*) and analyzed by flowcytometry. Killing by ^51^Cr release was assessed by 4 h coculture between the CL3 TCR^+^CD8^+^ T cell line and CaPo13 cells with or without anti-MHC class I (HLA-A, B, C) or anti-MHC class II (HLA-DR) mAbs as described previously. Effector/target (E/T) ratio ranged between 100∶1 and 1∶1.

## Supporting Information

Table S1 N-region specific probes for oligotyping analysis.
(0.30 MB TIF)
