# Correction: An Efficient Strategy to Induce and Maintain *In Vitro* Human T Cells Specific for Autologous Non-Small Cell Lung Carcinoma

**DOI:** 10.1371/annotation/df559170-4836-4bc7-a16d-44c7c6339cda

**Published:** 2011-01-13

**Authors:** Glenda Canderan, Paola Gruarin, Daniela Montagna, Raffaella Fontana, Gabriele Campi, Giulio Melloni, Catia Traversari, Paolo Dellabona, Giulia Casorati

The authors wish to add Gabriele Campi as the 5th author to the byline. Dr. Campi's affiliation is: Experimental Immunology Unit, Division of Immunology, Transplantation and Infectious Diseases (DIBIT), San Raffaele Scientific Institute, Milano, Italy. The correct byline is: Canderan G, Gruarin P, Montagna D, Fontana R, Campi G, Melloni G, Traversari C, Dellabona P, Casorati G. The correct citation is: Canderan G, Gruarin P, Montagna D, Fontana R, Campi G, et al. (2010) An Efficient Strategy to Induce and Maintain In Vitro Human T Cells Specific for Autologous Non-Small Cell Lung Carcinoma. PLoS ONE 5(8): e12014. doi:10.1371/journal.pone.0012014

