# Supplementary figures and images for: An Efficient Strategy to Induce and Maintain In Vitro Human T Cells Specific for Autologous Non-Small Cell Lung Carcinoma
Source: PLoS One. 2010 Aug 9;5(8):e12014. doi: 10.1371/journal.pone.0012014 (PMC2918513; doi:10.1371/journal.pone.0012014)

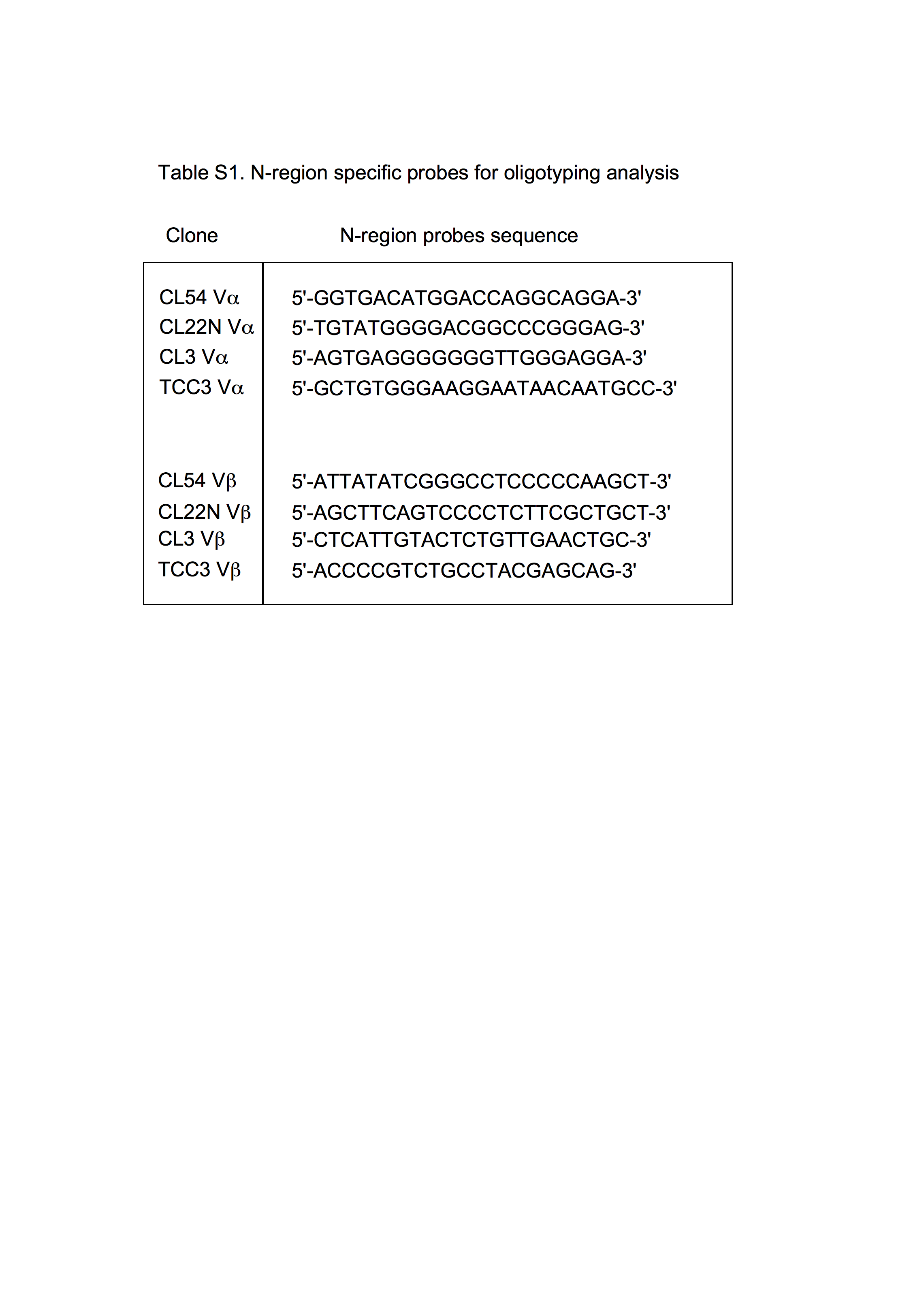

Supplement: Table S1 — N-region specific probes for oligotyping analysis. (0.30 MB TIF) [file pone.0012014.s001.tif]
